# On the Tribological and Oxidation Study of Xanthophylls as Natural Additives in Castor Oil for Green Lubrication

**DOI:** 10.3390/ma14185431

**Published:** 2021-09-19

**Authors:** Karla J. Moreno, María Teresa Hernández-Sierra, José E. Báez, Eloy Rodríguez-deLeón, Luis Daniel Aguilera-Camacho, J. Santos García-Miranda

**Affiliations:** 1 Department of Mechanical Engineering, National Technology of Mexico in Celaya (TecNM), Celaya 38010, Mexico; karla.mb@celaya.tecnm.mx (K.J.M.); daniel.ac@celaya.tecnm.mx (L.D.A.-C.); santos.gm@celaya.tecnm.mx (J.S.G.-M.); 2 Department of Chemistry, University of Guanajuato, Guanajuato 36050, Mexico; jebaez@ugto.mx; 3 Faculty of Chemistry, Autonomous University of Querétaro, Querétaro 76010, Mexico; eloy.q22@gmail.com

**Keywords:** xanthophylls, antioxidant additives, castor oil, sliding friction, wear, oxidation behavior

## Abstract

The present study focuses on an introductory analysis of the use of three xanthophylls as additives for green lubricant applications. For this purpose, the additives were characterized by FTIR and ^1^H-NMR techniques, and the bio-lubricants were described by their physical properties. The effect of the natural compounds on the friction and wear properties of bio-lubricants were evaluated by sliding friction tests under boundary conditions, as confirmed by an analysis of the lubricating film thickness. The antioxidant capacity was analyzed by FTIR spectroscopy. It was observed better wear protection in castor oil with xanthophylls than without these additives. The wear rate was reduced up to 50% compared with neat oil. Lesser beneficial effects were appreciated in friction coefficient since it was increased 25%. The best contribution was observed with astaxanthin as an additive. In addition, a significant improvement in the oxidation of castor oil, complemented with this additive, was exhibited by FTIR analysis. It was found that xanthophylls could be employed as additives for totally biodegradable lubricant applications since they have better tribological and antioxidant behavior than current additives.

## 1. Introduction

There is growing attention to the use of biodegradable lubricants to contribute to conserving the planet’s ecosystems [1]. This has triggered diverse research on numerous eco-friendly lubricants using different green bases and additives to improve lubrication properties in mechanical systems, as well as in engineering processes [2,3]. At this point, plant oils seem to be advantageous candidates for biodegradable bases since they show great improvements over petroleum products. They have higher lubricity and viscosity, lower volatility, greater shear stability, and high viscosity indexes (VI), as well as better biodegradability and toxicity properties [4]. However, the presence of the acyl group in their molecules and the unsaturated nature of most of their fatty acids provide vegetable oils with comparatively poor oxidative and thermal stabilities [5]. For this reason, to improve such weaknesses, the incorporation of additives or chemical modifications has commonly been resorted to [4]. Typical additives in the formulation of biolubricants consist of antioxidants, metal deactivators, corrosion inhibitors, extreme-pressure and anti-wear additives, pour-point depressants, and viscosity index improvers [6]. The total concentration of lubricant additives varies from a few hundredths of a percent to 30% or more [7]. The use of additives has been the main option for industrial applications [7]; nevertheless, not only do lubricant properties need to be upgraded, but they also must not contain metals and avoid the ecotoxicological problems caused by traditionally employed synthetic substances, such as zinc dialkyldithiophosphates, metal-containing dithiocarbamates, and chlorinated products [6].

Castor oil, which is extracted from the seeds of the *Ricinus Communis* L. plant, presents a great capacity to be used as a lubricant base oil. Its peculiar composition is rich in ricinoleic fatty acid (approximately 90 wt.%), an unsaturated omega 9 fatty acid with a hydroxyl group on its twelfth carbon, which makes it a natural oil with a high viscosity and excellent lubrication properties. Furthermore, it is an inedible oil that is easy to produce, which does not affect the global oil production for human consumption and allows the use of arid areas for its cultivation [8]. Castor oil, without additives, has shown very low friction coefficients under most lubrication conditions [9], but when it operates in boundary lubrication, wear tends to increase [10]. Additionally, due to its triglyceride nature, it still has insufficient oxidative and thermal behavior, which limits its service life in storage and industrial applications.

Some additives, both natural and synthetic, have been added to castor oil with the aim of increasing its antioxidant capacity or its friction and wear protection, exhibiting positive findings. For instance, Quinchia et al. (2011) [11] evaluated the oxidative stability of castor oil blended with (+)-α-tocopherol (TCP), propyl gallate (PG), and l-ascorbic acid 6-palmitate (AP) as natural antioxidant additives, and with 4,4′-methylenebis(2,6-di-tert-butylphenol) (MBP) as a synthetic antioxidant. Their results showed that the most effective biodegradable antioxidants were PG and MBP, whereas the TCP and AP were not effective. Later, Kumar et al. (2016) [12] evaluated some physical properties and the antioxidant capacity of castor oil with different volume fractions (0% to 1%) of butylated hydroxy toluene, butylated hydroxy anisole, and gallic acid as antioxidants. They found that castor oil, without and with antioxidants, has the potential to replace mineral oil. On the other hand, regarding the utilization of additives for the improvement of friction and wear, nanoparticles have gained relevance in the last few years. Wang et al. (2018) [13] studied the friction and wear performance of castor oil with the addition of hexagonal boron nitride nanoparticles (hBN) and found prominent reductions in friction and wear with 1 wt.% and 5 wt.% of hBN, respectively. Similarly, Singh, et al. (2020) [14] investigated the friction and wear properties of castor oil with TiO_2_; under the tested conditions, they principally found notable reductions in friction and wear with a concentration of 0.2 v% TiO_2_ nanoparticles. Karthikeyan et al. (2021) [15] analyzed the addition of molybdenum disulfide (MoS_2_) nanoparticles to castor oil without adding surfactants or property modifiers. They found that the tribological performance of nano lubricant was comparable to that of mineral oil, SAE20W40 multigrade engine oil. However, previous studies were performed separately for specific substances, not considering the effect of the operation and useful life properties together, and, in some cases, leaving aside the biodegradability properties of the additives.

Vegetable oils contain some natural antioxidants like polyphenols, tocopherols, carotenoids, chlorophyll, and δ–oryzanol [16], but the amounts thereof are not enough to allow the good oxidative capacity required for their use as industrial lubricants. Carotenoids are natural fat-soluble compounds that are made up of multiple isoprene units with a substituted and unsaturated cyclohexane ring at each termination [17]. Xanthophylls are a sub-family of carotenoids that contain oxygen in their terminal rings and that have the highest antioxidant potential [18]. Carotenoids have principally been used as antioxidants and/or coloring agents in the food, pharmaceutics, and cosmetic industries. Concerning antioxidant properties, carotenoids are efficient at scavenging singlet molecular oxygen and peroxyl radicals [18]. The influence of carotenoids on edible oil oxidation has been studied and it has been demonstrated that they have outstanding results when compared with synthetic additives [18]. However, scarce applications in the industrial sector have been reported.

For the above reasons, this paper presents a preliminary study on the use of three xanthophylls (lutein, zeaxanthin, and astaxanthin) as additives in castor oil to improve its performance and oxidation properties for lubricant applications in mechanical systems. To do this, the effect they have on the friction and wear behavior of castor oil is first analyzed using standardized methods. Subsequently, the response to oxidation is evaluated by monitoring the primary and secondary oxidation products of castor oil using infrared spectroscopy techniques.

## 2. Materials and Methods

### 2.1. Preparation of Bio-Lubricants

**Figure 1:**
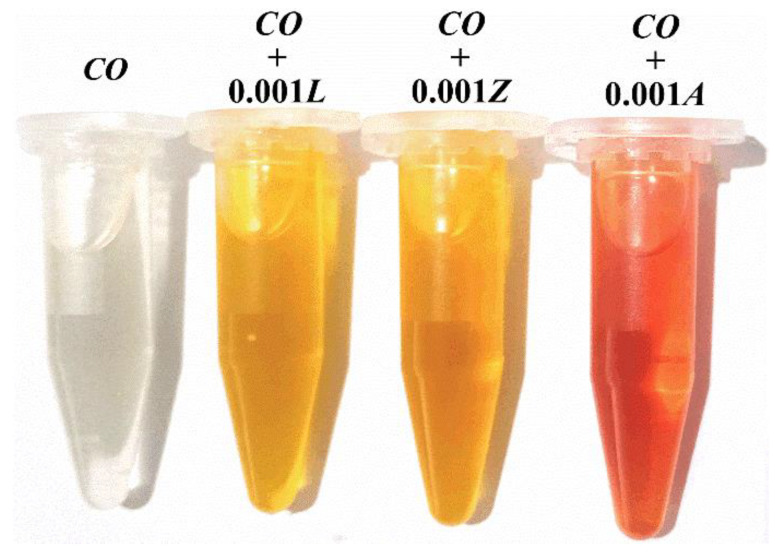
CO with a concentration of 0.001 molar of xanthophylls as additives: lutein (L), zeaxanthin (Z), and astaxanthin (A).

For the formulation of bio-lubricants, lutein (L), zeaxanthin (Z), and astaxanthin (A) were selected as lubricant additives because they are among the most common xanthophylls, they are abundant, and have great antioxidant capacities. The additives were obtained from the native Mexican flower (*Tagetes erecta* L.), commonly called Cempasúchil. The extraction was made using a simple, efficient, and low-cost method, reported previously in [17]. Then, powders of the natural additives were incorporated into neat castor oil (*CO*) which was acquired from Abreiko, Guadalajara (Jalisco), Mexico. It was reported that castor oil contains a natural concentration of some carotenoids of about 2.05 mg/kg [16]. In this study, a molal concentration of 0.001 (approximately 568 mg/kg) of each xanthophyll was fixed, based on a previous laboratory solubility test. This amount of additive was between the typical concentration ranges of additives established in the literature [19]. Bio-lubricant formulations of a homogeneous consistency were obtained after heating the mixtures to 80 °C with constant magnetic stirring (100 rpm) for one hour. The miscibility of each xanthophyll in castor oil was corroborated by centrifuging each bio-lubricant at 5000 rpm for 10 min and no precipitation was observed. Furthermore, no solid particles were observed ten months later. Figure 1 illustrates a photograph of the appearance of neat castor oil and the bio-lubricant formulations. As expected, it was observed that the lubricating solutions showed a different color according to the characteristic color of the employed additive.

### 2.2. Chemical Characterization of Additives

The natural additives were characterized by attenuated total reflection Fourier transform infrared (ATR-FTIR) and nuclear magnetic resonance (^1^H-NMR) spectroscopies. ATR-FTIR spectra were recorded with a Spectrum 100 spectrometer from Perkin Elmer, Waltham (MA) USA with a LiTa03 detector and KBr beam splitter. A ZnSe attenuated total reflectance device was employed, and the analysis was done from 650 to 4000 cm^−1^ with a resolution of 4.0 cm^−1^. On the other hand, for the ^1^H-NMR analysis, additives were diluted in chloroform-d (CDCl_3_). Spectra were recorded using a Bruker Avance III HD 500-MHz spectrometer from Bruker, Billerica (MA) USA at room temperature and were referenced to the residual solvent at 7.3 δ (ppm).

### 2.3. Physical Characterization of Bio-Lubricants

The physical properties of lubricants play a key role in their operational lubrication performance. Therefore, in this work, the density, viscosity, viscosity index, and pressure–viscosity coefficient of bio-lubricant formulations were studied. The density and kinematic viscosity at 40 °C and 100 °C were determined using a pycnometer Cannon Instrument, State College (PA), USA (ASTM D 891), and glass capillary viscometers from Cannon Instrument, State College (PA), USA (ASTM D445) method, respectively. A thermal bath with 0.1 °C precision was employed to control the temperatures of samples for these tests. Then, the viscosity index was calculated from the obtained viscosity values of bio-lubricants, according to the ASTM D 341 standard. Additionally, in order to have an estimation of the lubricant film thickness and the lubrication regime, the pressure–viscosity coefficient was evaluated from the bulk properties of the lubricants as per a reported procedure [9].

### 2.4. Lubricating Regime Estimation

The friction and wear behavior of a mechanical system strongly depends on the thickness of the lubricating film between the surfaces that are in contact, and, therefore, in the lubricating regime. To define and comprehend the tribological properties of the new bio-lubricants, the film thicknesses and lubrication regimes were estimated. The methodology employed was based on the Hamrock and Dowson theory [20], following the method described in [9].

### 2.5. Tribological Performance Evaluation

The tribological performance of the lubricant preparations was evaluated using sliding friction tests under boundary lubrication conditions. These experiments were accomplished using a tribometer from CSM Instruments, Needham (MA), USA with a ball-on-disk configuration, based on the ASTM G-99 standard. For this evaluation, disks of AISI 4140 steel were used because this material is one of the most employed steel in the industry due to its notable mechanical properties [21]. Whereas commercial balls of tungsten carbide (WC) were employed as counterparts. The disk and pin materials were cleaned with methanol prior to being tested to avoid any contamination. Table 1 lists the characteristics and properties of these materials.

**Figure 2:**
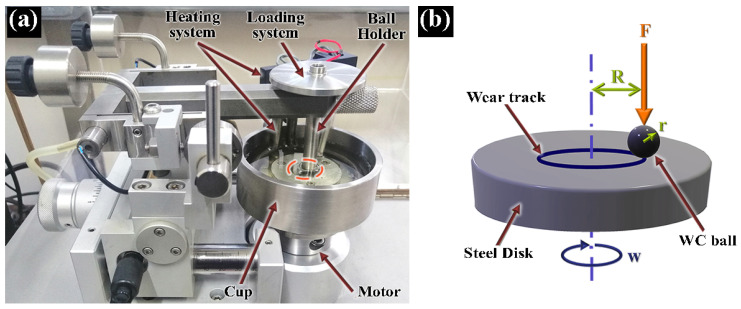
High temperature lubricated friction tests: setup (**a**), and ball-on-disk scheme (**b**).

During the experiments, a normal load of 1 N (mean contact pressure of 900 MPa) was applied through the static counterpart located 2 mm from the center of the disk, while the latter was rotated at a linear speed of 0.025 m·s^−1^ (750 rpm). The materials were completely immersed in 60 mL of each lubricating medium, which was maintained at a temperature of 100 °C utilizing an electrical resistance. It is important to mention that, since the lubricating method was immersion, the amount of wear particles in the oil were not controlled during the tests. Room humidity and temperature were also controlled at 45% and 25 °C, respectively. Figure 2a shows the high-temperature lubricated test arrangement. In this configuration, the cup served to hold the steel disks, transmit the rotational movement, as well as to contain the lubricating medium. In this figure, the contact zone between the ball and disk is highlighted by a red circle. Figure 2b describes the general ball-on-disk configuration. The wear track illustrated in this figure corresponds to the wear mark generated on the disk surface when the ball counterpart has a considerably higher hardness than the disk material.

The evolution of the kinetic friction coefficient (*µ_k_*) was recorded through the tests by the computer of the equipment which also provides the average values. After completing 30,000 cycles, the worn surfaces were analyzed by optical microscopy with Carl Zeiss Axio Imager.A1m microscope from Carl Zeiss, Oberkochen (Baden-Württemberg), Germany. The wear track widths were measured, and the volume loss (V) and wear rate (K) were calculated according to Equations (1) and (2), respectively. In this study, only the wear rate values are reported.

V = πRd^3^/6r (1)

K = V/FS(2)

In the previous equations, R represents the wear track radius, d the wear track width, r the ball radius, F the normal load, and S the total sliding distance that is calculated by S = 2πRC where C is the number of cycles.

### 2.6. Oxidation Performance Evaluation

Oxidation is a degradative process in lipids that occurs when they are exposed to light, heat, oxygen, enzymes, metals, metalloproteins, and microorganisms. Consequently, oxidation can be accomplished via different routes [22], and even through a synergy of several routes. Autoxidation and thermal oxidation of unsaturated fatty acids, which are the most common oxidation pathways, occur through a well-known free radical-mediated chain reaction with three stages: initiation, propagation, and termination [22]. It has been identified that hydroperoxides are the primary oxidation products of lipids, and they then decompose into secondary products, such as aldehydes, ketones, alcohols, hydrocarbons, volatile organic acids, and epoxy compounds. In this way, the measurement of these products, as well as that of free radicals, gives a size of the degree of deterioration of lipids by oxidation [22]. Fourier transform infrared spectroscopy is an easy, fast, and relatively inexpensive technique that can be used to analyze the oxidative degradation of vegetable oils with a good correlation among conventional methods [22,23]. Effective results have been found with ATR-FTIR spectroscopy when evaluating the entire mid-infrared spectrum for identification between acceptable, marginal, and unacceptable frying oils, determined according to their oxidation end products [23]. 

In this contribution, the effects of xanthophylls as natural additives in castor oil oxidation were evaluated by attenuated total reflectance mid-infrared spectrometry using the equipment defined earlier. Analyses were performed by comparing the bands between 3800 and 3150 cm^−1^ corresponding to O–H stretching absorption to evaluate the formation of hydroperoxides (primary oxidation products), as well as by the carbonyl band (C = O) from 1800 to 1630 cm^−1^ where the IR absorption of oxidation subproducts, such as aldehydes, ketones, or acids, are located [22]. This evaluation was accomplished for all bio-lubricant formulations at the beginning of the friction tests, and after 10,000, 20,000, and 30,000 elapsed cycles. For this reason, after a certain number of operating cycles, representative aliquots of bio-lubricants were placed on a clean ATR plate and scanned from 650 to 4000 cm^−1^. The area of the bands to be analyzed was calculated from the absorbance spectra using equipment software in the corresponding wavenumber ranges. The band area or peak area is a term in spectroscopy quantitative analyses. It is calculated by integrating across peak width, and, frequently, it is found to be more accurate than peak-height measurements [24].

## 3. Results

### 3.1. Chemical Characterization of Additives

**Figure 3:**
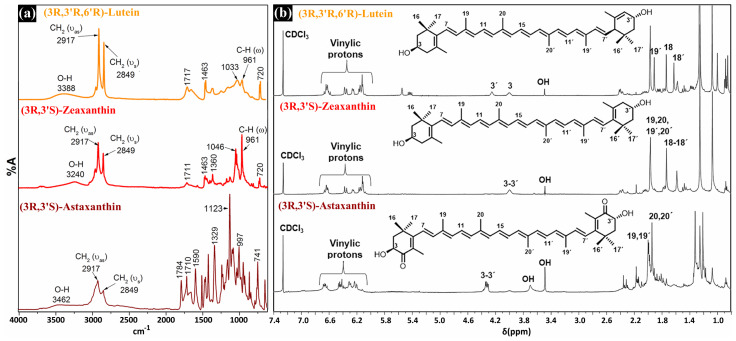
ATR−FTIR (**a**) and ^1^H−RMN (**b**) spectra of xanthophylls.

Figure 3 shows the ATR-FTIR and ^1^H-NMR spectra of xanthophylls. These spectra were comparable with those previously reported for lutein [25], zeaxanthin [26] and astaxanthin [27]. In Figure 3a, it can be seen that the characteristic bands of these carotenoids only showed differences in shape, shift, and intensity. The broad bands between 3200 and 3400 cm^−1^ were attributed to the hydroxyl groups, and the stretching vibrations at 2917 and 2849 cm^−1^ to the aliphatic CH_2_ groups. On the fingerprint region of lutein and zeaxanthin, relatively strong bands at about 961 cm^−1^ can be observed, which corresponded to the out of plane C–H deformation, with bands at about 1046 cm^−1^ to the C–O in the stretching mode. The molecular structures of the xanthophylls were corroborated by ^1^H-NMR analyses, as shown in Figure 3b, which represent the most important signals. For lutein the signal H-18′ was situated at 1.63, H-18 at 1.74, H-19′ at 1.91, H-3 at 4.01, and H-3′ at 4.25 δ (ppm). In zeaxanthin H-18 and H-18′ they were observed at 1.74, H-19, H-19′, H-20, and H-20′ at 1.97; and H-3 and H-3′ at 4.00 δ (ppm). Finally, for astaxanthin H-20 and H-20′ were situated at 1.99, H-19 and H-19′ at 2.00, and H-3 and H-3′ at 4.33 δ (ppm). In all cases, the OH proton was located at 3.5 δ (ppm) and the vinylic protons between 6.0 and 6.7 δ (ppm). A detailed description of all signals can be found in [17].

### 3.2. Physical Characterization of Bio-Lubricants

Table 2 summarizes the physical properties of the bio-lubricants. It can be noted that the obtained values for physical properties of castor oil were closed to those reported in the literature [8,13,14]. However, the density of castor oil at 40 °C slightly increased with all additives concerning the neat oil. Whereas, at 100 °C, an opposite effect on the density was observed; this property was decreased to some extent. Moreover, the kinematic viscosity at 40 °C of bio-lubricants decreased up to 17% (*CO* + 0.001*L*) about castor oil without additives. At the highest temperature, lutein and zeaxanthin did not affect the viscosity of castor oil, but astaxanthin slightly increased this value. This effect was positive since generally an increase in viscosity at low temperatures turns out to be counterproductive; however, at high temperatures it is desirable. Although several authors have reported that small amounts of carotenoids produce changes in the viscosity of various products, such as oleogels [28] and emulsions [29], the mechanism is has not been well studied so far due to its complexity. Other additives had exhibited a slight increase in both the density and viscosity of castor oil. Some examples are butylated hydroxy toluene, butyalted hydroxy anisole, and gallic acid [12]; and nanoparticles of hexagonal boron nitride [13] and titanium dioxide [14]. This phenomenon could be due to the high density and concentration of the employed additives. 

On the other hand, the observed variations in the viscosity of the *CO*-xanthophyll bio-lubricants were reflected by a significant improvement in their viscosity indexes (VI), which were enhanced up to 30% (*CO* + 0.001*A*). The viscosity index of lubricants is a vital parameter to determine their general quality; the higher the viscosity index, the more stable the viscosity of a lubricant is in a greater range of temperatures. It is important to mention that this enhancement in VI is similar to that observed with TiO_2_ as an additive [14]. Considering the impact of xanthophylls on the pressure–viscosity coefficient, a reduction of up to 50% was observed (*CO* + 0.001*A*) concerning castor oil. The influence of viscosity and the pressure–viscosity coefficient on the creation of lubricant layers capable of supporting a load and protecting surfaces have been discussed previously [9]. 

### 3.3. Lubricating Regime Estimation

**Figure 4:**
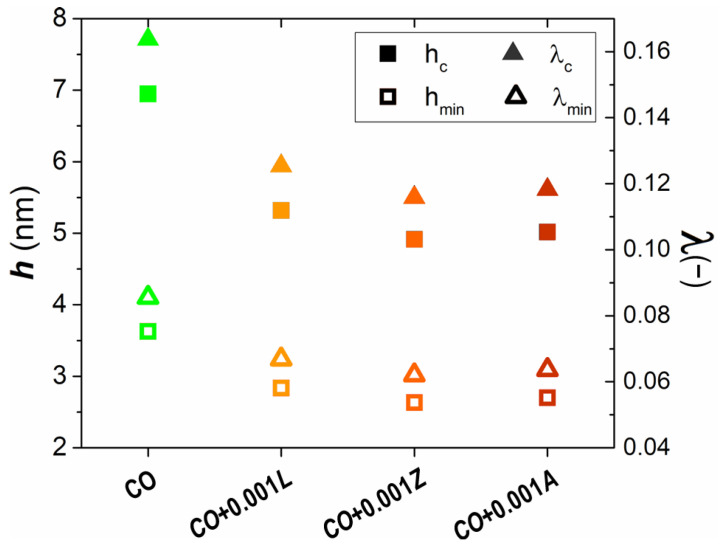
Central and minimum lubricant film (h) and lambda ratio (λ) of bio-lubricants.

Figure 4 shows the central and the minimum lubricant film thicknesses (h) and lambda ratios (λ) of bio-lubricants. As expected from the intended test conditions, it can be noted that the film thicknesses for all bio-lubricants were within the range of 2 to 8 nm. The addition of xanthophylls to castor oil decreased the thickness of the protective lubricant layer up to 30%. The bio-lubricant complemented with zeaxanthin was the most affected. The lambda ratio relates to the minimum film thickness and the surface roughness (σ) of the pin and disk materials, λ = h/√(σ_1_^2^ + σ_1_^2^) [10]. In turn, the λ parameter defines the lubrication regime in which a lubricated system operates. The system works by hydrodynamic lubrication when λ is higher than 3, in mixed lubrication when λ ranges from 1 to 3, and in boundary lubrication when λ is lower than 1 [9,10]. Therefore, the results in Figure 4 confirmed the boundary conditions for all lubricated systems.

### 3.4. Tribological Performance Evaluation

**Figure 5:**
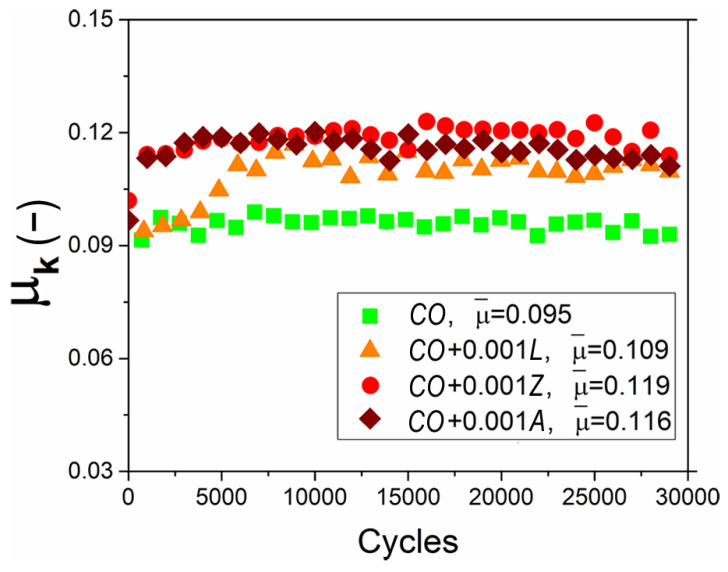
Effect of xanthophylls as additives in *CO* on the kinetic friction coefficient.

Figure 5 exhibits the influence of xanthophylls on the kinetic friction coefficient (µ_k_) of castor oil. It is important to note that, in all cases, friction coefficient results were within a narrow range, from 0.09 to 0.12. The system lubricated with castor oil without additives produced a constant friction coefficient of about 0.095 during the whole test; however, when organic additives were included, the friction coefficient increased up to 25%. In the starting cycles, the friction coefficient achieved with the addition of lutein showed a value near to that of the castor oil. This behavior could be because it helped to generate a thicker lubricating layer than the other additives. Nevertheless, after 5000 cycles, the friction coefficient reached and remained at an approximate value of 0.109. On the other hand, the behavior of the friction coefficients for lubricants with zeaxanthin and astaxanthin were very close. Although, that obtained with astaxanthin showed a greater stability and a lower value. All the friction coefficients agreed with the lubricating film thickness values, where the greater the thickness, the less friction was obtained.

**Figure 6:**
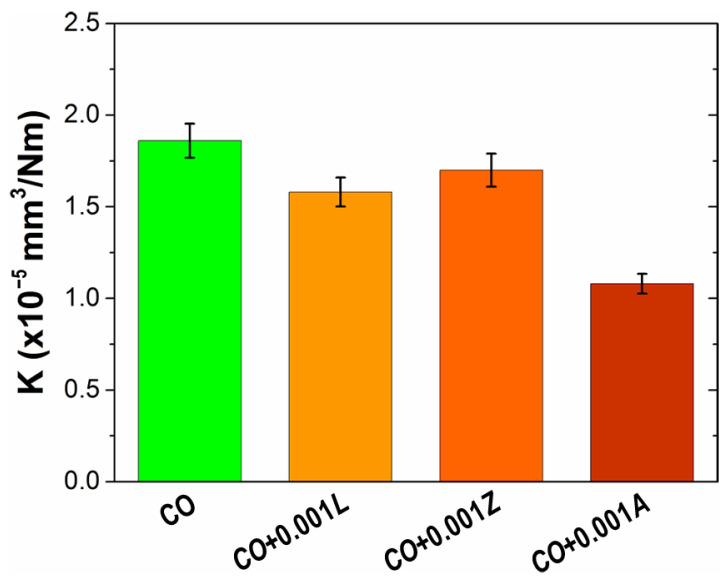
Effect of xanthophylls as additives in *CO* on the wear rate (K) behavior.

**Figure 7:**
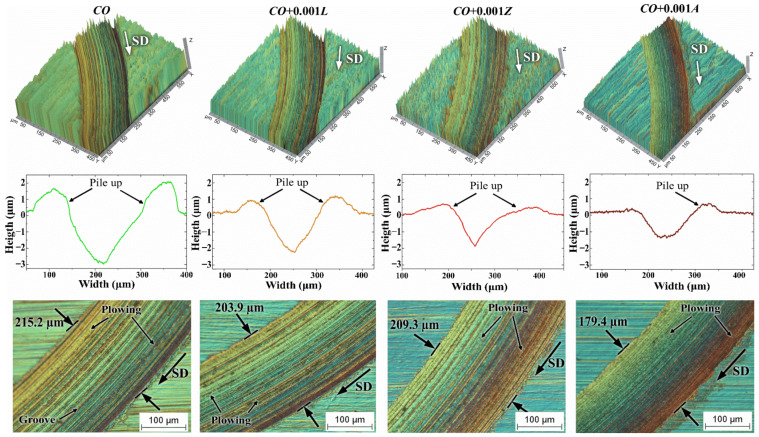
Effect of xanthophylls as additives in *CO* on the topography of worn surfaces and wear track width (micrographs at 200X). SD means sliding distance.

The effects of additives on wear behavior are presented in Figure 6 and Figure 7 using the wear rate values and via analyses of the worn surfaces, respectively. Contrary to the observed friction results, the addition of xanthophylls to castor oil demonstrated a notable improvement in wear protection. Figure 6 shows the wear rate values to quantify the wear response of the bio-lubricant formulations. The best contribution in wear was observed with incorporation of Astaxanthin at 0.001 molal. The mean wear rate value with this additive was 1.08 × 10^−5^ mm^3^/Nm, which was 42% lower than that achieved with castor oil without additives. The reductions in wear are quite significant for lubricant applications, since low wear rate values enhance the useful life of machine components. 

Figure 7 displays moderately evident wear due to the boundary conditions under which the bio-lubricants were tested. As the wear was produced by interactions of solid surfaces in contact and the surrounding environment, the high temperature of the lubricating medium, as well as the heat generated during friction between the two surfaces, contributed to cause greater damage to the materials in contact. It can be seen in Figure 7 that the topography of the worn surface changed according to the additive. The surface tested with castor oil without additives exhibited an average wear track width of 215 µm, generated by the abrasive wear mechanism as the groove and plowing marks suggested. On its wear profile, the wear mark was also deep and with a great pile-up height. This was because, during the abrasive wear, the substrate particles removed from the grooves slid over the substrate and were displaced into the pile-up around the wear track. The plowing wear presented when the displacement of the piled-up particles in the wear groove/ridge occurred without being removed from the sliding track. On the other hand, when using organic additives, the predominant wear mechanism was plowing type abrasion, where finer groove marks were observed, thus indicating higher wear resistance. The width and depth of the wear track decreased significantly with the addition of xanthophylls to castor oil. In addition, the piled-up material was reduced considerably. 

**Figure 8:**
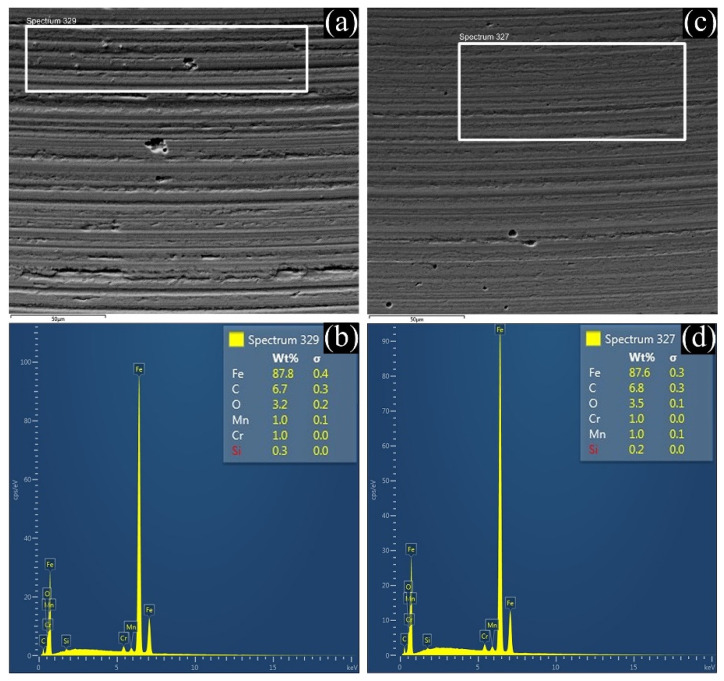
SEM and EDX analyses of steel surfaces tested with *CO* (**a**,**b**), and *CO* with astaxanthin (**c**,**d**).

In order to analyze tribofilms in the lubricated systems in depth, scanning electron microscopy/energy dispersive X-ray spectroscopy was employed to analyze the main components of the tribofilms. A scanning electron microscope (Quanta 3D 200i) from Thermo Fisher Scientific, Waltham (MA), USA equipped with an Oxford X-MaxN-50 EDX from Abingdon-on-Thames (Oxfordshire), UK, was employed. Figure 8 exhibits the SEM and EDX analyses on the worn surfaces lubricated with neat castor oil and castor oil with astaxanthin (CO+0.001Astx). Firstly, it can be seen that the samples tested with *CO*+0.001*A* showed smoother surfaces than the sample tested with *CO*, revealing less wear. Furthermore, in the EDX spectrum, a higher content of oxygen on the worn surface tested with this lubricant was observed, which could be related to the formation of better oxide films that prevent higher wear. Although there is also oxygen in the sample evaluated with *CO*, higher oxidation could increase the reactivity of the oxygen with the metal, exceeding the rate of removal of the protecting oxide layer [30].

**Figure 9:**
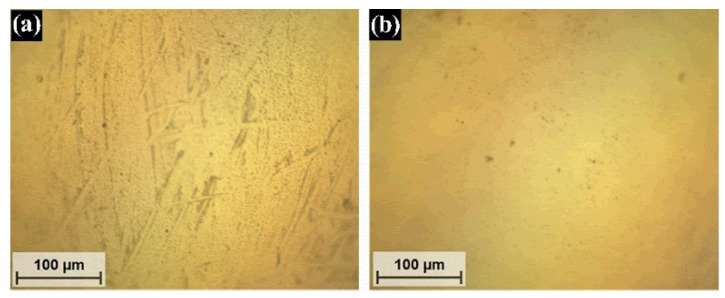
Micrographs (at 200×) of counterpart surfaces: tested (**a**) and untested (**b**).

Regarding the counterpart, it is important to mention that, in this work, the wear only occurred in the disk material since the counterpart (WC) is harder than the AISI 4140 steel disk. Figure 9 shows a representative micrograph of a tested and an untested counterpart. In Figure 9a, slight abrasive wear marks can be observed on the tested surface. Nevertheless, the quantification of wear is not possible at this level. Figure 9b exhibits an untested counterpart surface to compare the irrelevant damage.

Table 3 summarizes comparison data in recent works for trending additives in castor oil and the effects they have on friction and wear compared to oil performance without additives. Wang et al. [13] studied the friction and wear mechanisms of castor oil with the addition of hexagonal boron nitride nanoparticles (hBN), under low load and high-speed tests (10 min duration), their results exposed an increase in friction up to 22% when 8 wt.% hBN nanoparticles were added to castor oil in comparison with that of neat oil, but the wear track width decreased by 28%. Singh et al. (2020) [14] reported friction and wear characteristics of castor oil with titanium dioxide (TiO_2_) nanoparticles as additives. The authors found higher friction (about 12% increase) and wear (K on the order of 10^−4^ and nearly 11% higher) with castor oil + 0.3 v% of TiO_2_. In this work, the authors suggested that the problem was due to the agglomeration of the nanoparticles in oil with the increasing of the additive concentration. The study of Karthikeyan et al. (2020) [15] on the thermophysical and wear properties of eco-friendly nano lubricants, also revealed an increase of 12.5% in friction when 0.7 wt.% MoS_2_ nanoparticles were employed as additives in castor oil. In the present study, an additional comparison was performed with one of the most employed synthetic anti-wear additives to castor oil. A commercial zinc dialkyl dithiophosphate (ZDDP) additive from Rev X Products Inc, Grand Rapids (MI), USA was employed at the recommended concentration by the producer. The new lubricant was tested by evaluating the same tribological conditions of the performance of this additive in Castor oil. It was observed to have a beneficial effect on the friction behavior, since the friction coefficient was reduced by 23%; however, this additive increased the wear rate by 31%.

ZDDP usually has good antioxidant and anti-wear capacities as it forms a protective layer due to the chemical reaction between its components and metal surfaces. However, this may have different responses depending on the system in question, according to the concentration, speed, and the type of base lubricant, among other characteristics [31]. At low temperatures and high speeds, for example, Kumar et al. (2019) [32] found an increase in friction with the addition of ZDDP to refined soybean oil, which was attributed to the formation of thicker layers of ZDDP. Positively, the thicker film helped to reduced wear. On the other hand, at a higher temperature of 100 °C, Clancy (2013) [31] showed that ZDDP additive has a negative effect on the friction and wear response of cruciferae oils since ZDDP does not greatly affect the tribofilm naturally formed by vegetable oils. Only with mineral oil base did the ZDDP exhibit good results, however. In the present study, the same phenomenon could occur; however, as castor oil is recognized as one of the most efficient vegetable oils for reducing friction due to its hydroxyl polar group, the combination with ZDDP film could improve friction performance. Such a film however was not capable of protecting against wear. Further work could be performed to find the most favorable conditions for using ZDDP as an additive in castor oil.

As stated above, despite operating in the boundary lubricating regime where friction and wear are greatly increased, and even during a much longer test durations (252 min), the use of xanthophylls as additives in castor oil helped to reduce wear by up to 42% and presented a maximum increase in friction by 25% concerning neat oil. This comparative analysis denoted that the response to friction and wear of xanthophylls as additives is very comparable to that of other types of additives, even in high-level severe conditions. Furthermore, xanthophylls are natural oil-soluble compounds and do not alter the ecological balance of our planet, which gives them viability to be used in completely biodegradable lubricants.

### 3.5. Oxidation Performance Evaluation

**Figure 10:**
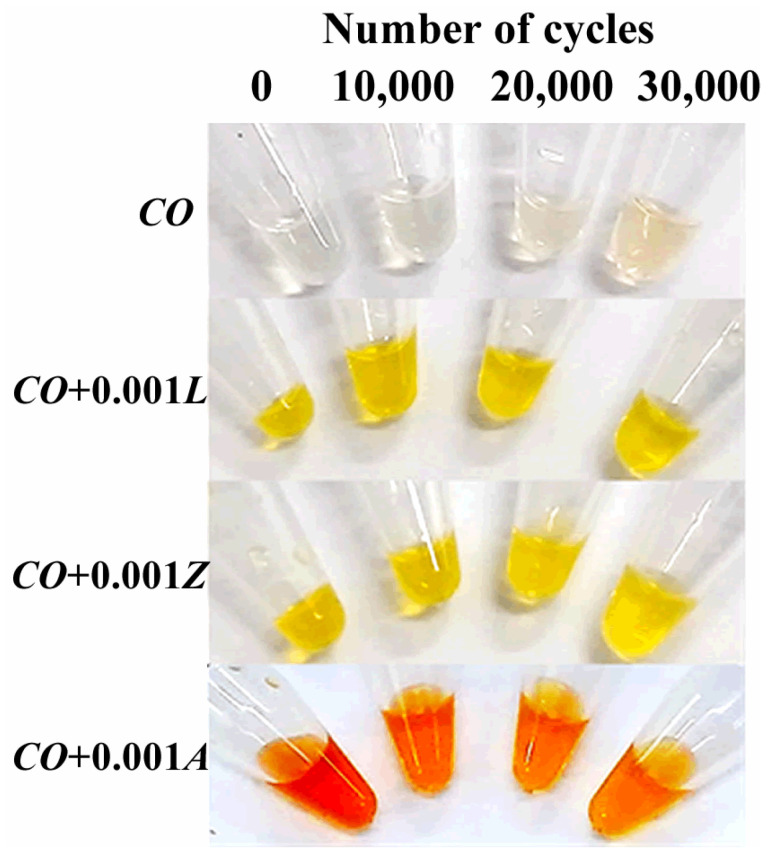
*CO* with different xanthophylls during lubricated friction tests.

The low oxidative stability of castor oil is mainly due to the presence of the double bond and the hydroxyl groups on its main fatty acid, which are easy to oxidize and yield a broad range of oxidation products [33,34]. On the other hand, carotenoids are extremely susceptible to degradation by exposure to light, high temperatures, and oxygen due to their highly unsaturated and non-polar molecules. Therefore, it is expected that xanthophylls may act as sacrificial oxidative substances (due to their consumption) decreasing castor oil oxidation. As lubricant deterioration can be indirectly qualified by the change in color [7], in Figure 10 the appearance of castor oil with different xanthophylls as additives after certain lubrication cycles is exposed. Firstly, it can be observed that as the number of cycles increased, the color of castor oil without additives became darker, revealing the oil deterioration. This could be caused due to the creation of high-molecular-weight oxidant products that provide intensive light absorption and scattering effect. On the other hand, a slight decrease in the characteristic color of each bio-lubricant formulation was observed, which may be due to additive consumption during friction and wear experiments.

**Figure 11:**
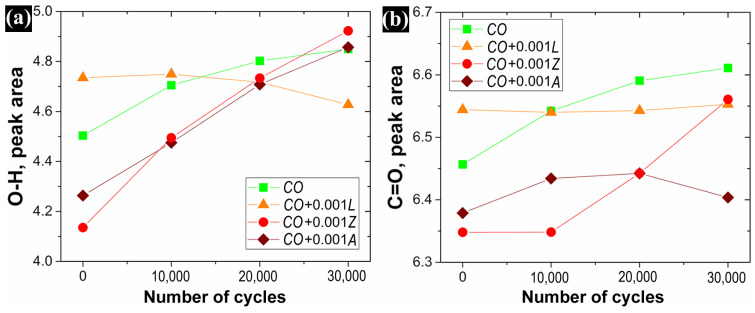
Effect of xanthophylls in *CO* over its hydroxyl and carbonyl groups signal.

The effect of xanthophylls as lubricant additives in castor oil oxidation is presented in Figure 11 by the change in the hydroxyl and carbonyl group signal areas. The increment in the area of the characteristic O–H stretching and C = O absorption bands could be attributed to the generation of hydroperoxides as primary oxidation products, and to the creation of volatile and non-volatile chemical products originated from peroxides decomposition (secondary oxidation compounds), respectively [22]. At the initial stages, zeaxanthin and astaxanthin notably improved the oxidation properties of castor oil by displaying the smallest values of O–H and C = O peak areas compared to oil without additives. Regarding lutein, at this stage and concentration, it exhibited a pro-oxidant effect allowing a higher creation of oxidation products in the castor oil. On the other hand, at further stages, the evolution of the hydroxyl group area tended to decrease while the carbonyl band remained almost constant, and even slightly increased at 30,000 cycles. This suggested that lutein showed better antioxidant capacities while concentration is reduced due to consummation. Different behavior was observed with the addition of zeaxanthin and astaxanthin to castor oil, of which the O–H bands tended to increase at higher numbers of operating cycles. However, as the rate of hydroperoxides formation outweighs the rate of decomposition during the initial phase of oxidation [22], the C = O band with Zeaxanthin tended to increase at larger stages, reaching almost the same value as that obtained with lutein. Astaxanthin slightly increased the C = O band at further stages with a tiny decline at 30,000 cycles but much lower than the others. Lubricants need to possess good oxidation stability to increase the useful life of lubricant oil. A comparison with other antioxidative studies is difficult due to the complexity of the oxidation mechanisms of both the oils and the molecules studied, as well as because the results vary according to the methodology employed [35]. Despite this, the present results suggest that astaxanthin as an additive in castor oil has better antioxidant capacity than lutein and zeaxanthin, which was also observed by Lee and Min [36].

**Figure 12:**
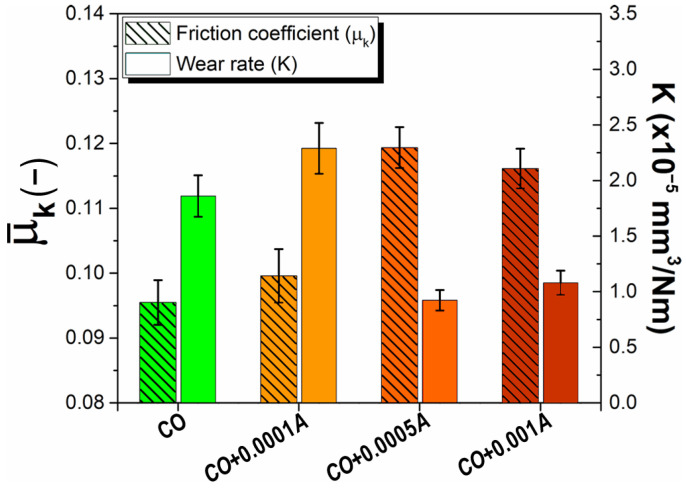
Effect of different concentrations of Astaxanthin as an additive in *CO* on the friction (µ_k_) and wear (K) response.

To evaluate the influence of additive concentration and to find possible amounts of additives that could improve friction and wear performance, new tribological experiments were performed. Smaller amounts of astaxanthin were studied using tribological tests because of the better wear and oxidation performance exhibited. Figure 12 incorporates the friction and wear behavior of the new biodegradable astaxanthin–castor oil lubricant formulations. It can be observed that, as additive concentration increases, the friction coefficient also increases by 4% and 25%, presenting a slight decrease at the maximum concentration (22%). Wear behavior shows a maximum wear rate at the minimum concentration of astaxanthin (K = 2.29 × 10^−5^ mm^3^/Nm); then, wear significantly decreased at the intermediate concentration (K = 9.23 × 10^−6^ mm^3^/Nm) resulting in a 50% reduction in wear in comparison to zero concentration of astaxanthin (K = 1.86 × 10^−5^ mm^3^/Nm). Finally, the wear slightly increased at the highest concentration (K = 1.08 × 10^−5^ mm^3^/Nm) but this is still much better than the one obtained without additives. The highlight of these results is that they suggest that a more favorable concentration of Astaxanthin could be found between 0.0001 and 0.0005, where there is a turning point in both friction and wear behavior. This point would be worth studying in the future. Thus, it can be stated that a critical concentration of other additives should be inherent to them.

## 4. Discussion

**Figure 13:**
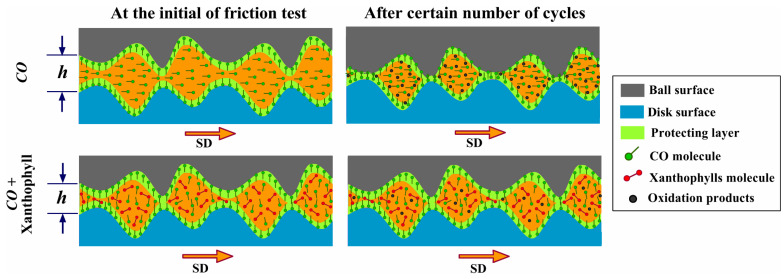
Schematic diagram of the friction and wear mechanism of xanthophylls as lubricant additives in *CO*. SD means sliding distance.

In the boundary lubrication regime, friction tends to be higher than in any other regime because there is greater contact between the surfaces, and the lubricant film is trapped between their roughnesses. The smaller the thickness of the protective layer, the greater the friction generated. This is one important factors that could influence the friction response of bio-lubricants. Since the addition of xanthophylls to castor oil decreased the pressure–viscosity coefficient significantly, the lubricant film thickness was decreased, and thus a higher friction was generated. The thickness of a boundary lubricating film can be only a few molecular layers thick (1–10 nm) [37], like the observed values of this study. In this state, the friction and wear performance depend on the physicochemical characteristics of the lubricant base and the additives with the surfaces. Here, the protective boundary film is, molecularly, a thin layer of lubricant and surface reactive additives. At this point, it had been established that molecules with longer and polar chains will favor the thickness of the adsorbed film and enhance surface interactions, thus reducing friction [38]. This could also benefit the low friction obtained with castor oil lubrication, which has a long and polar chain structure. Nevertheless, when xanthophylls were added, as they have different non-polar molecular structures with an unsaturated cyclohexane ring at the ends, and less effective molecules in reducing friction, consequently friction coefficients were increased. High-temperature tribological processes are very complex because they involve changes in the properties of both, materials, and lubricants. Materials often exhibit changes in the mechanical properties and the chemical and morphological characteristics of their surfaces due to oxidation and diffusion caused by adhesive/abrasive wear and thermal fatigue [39]. All above is represented by a schematic diagram of the friction and wear mechanism of xanthophylls as lubricant additives in castor oil and is depicted in Figure 13. 

On the other hand, lubricant oxidation is crucial for the tribological performance of a mechanical system. In the short term, the effect of lubricant oxidation can provide better lubricity since viscosity tends to slightly increase. However, in the long term, when lubricant degradation has reached extreme levels, it can result in a significant reduction in lubricating capacity, inducing sediment and sludge formation, corrosion, as well as material degradation [40]. Comparing the SEM images in Figure 8, it can be seen that the effect of corrosive wear was also diminished with the addition of astaxanthin. For that reason, this characteristic could be the key factor that defined the potential of these xanthophylls as lubricant additives. Given that they helped to reduce the degradation of the lubricant by oxidation, the wear damage on surfaces decreased considerably. Astaxanthin, which was the additive that most improved the antioxidant capacity of the oil, was also the one that provided greater protection against the wear of materials. 

## 5. Conclusions

This paper presented a preliminary study of a fully bio-lubricant of castor oil with xanthophylls: lutein, zeaxanthin, and astaxanthin, as antioxidant additives.

A concentration of 0.001 molal of the studied Xanthophylls produced the following results:

- The viscosity index of castor oil was increased with the incorporation of all xanthophylls. An improvement up to 30% was reached with astaxanthin in *CO* compared with the neat oil.
- The lubricant film thickness of bio-lubricants decreased to nearly 30% in comparison to the neat oil, inducing an increase in friction up to 25%. The highest friction coefficient (µ_k_ = 0.119) was obtained with the zeaxanthin additive.
- The wear protection capacity of castor oil was enhanced with the addition of all xanthophylls. The wear rate (K) was decreased up to 42% with the bio-lubricant added with astaxanthin in comparison with that of the neat castor oil.
- The oxidation behavior of castor oil was notably enriched using xanthophylls as additives. The best antioxidant capacity was observed in the astaxanthin additive.

The wear protection of castor oil was improved by up to 50% with lower concentrations of astaxanthin (at 5 × 10^−4^ molal), comparing to the *CO* without additives. A possible virtuous astaxanthin concentration, where a better balance of friction and wear behavior could be presented, was found to be between 1 × 10^−4^ and 5 × 10^−4^ molal.

In terms of balance, the better performance of xanthophylls compared with other current additives, as well as their organic nature, suggests they could be employed as additives for lubricant applications with a lower negative impact on the environment.

Further research will be centered on a complete analysis of the antioxidant capacity of xanthophylls in castor oil, as well as in the optimization of the concentration of these lubricant additives.

## Figures and Tables

**Table 1:** Characteristics and properties of the materials for tribological tests.

Material	Dimensions (mm)	Surface Roughness, Ra (µm)	Vickers Hardness (GPa)	Elasticity Modulus, E (GPa)	Poisson’s Ratio
AISI 4140 Steel Disks	25.4 × 5 (d, t)	0.03	1.9	210	0.27
WC balls	3 (d)	0.03	16.7	650	0.22

d: diameter, t: thickness.

**Table 2:** Physical properties of biolubricants.

Physical Property	Temperature, °C	*CO*	*CO* + 0.001*L*	*CO* + 0.001*Z*	*CO* + 0.001*A*
Density (kg/m^3^)	40	945	950	949	949
	100	925	919	913	911
Kinematic viscosity (mm^2^/s)	40	265	221	226	231
	100	22	22	22	24
Viscosity index, VI	--	100	120	118	130
Pressure–viscosity coefficient (GPa^−1^)	100	48.7	29.1	26.0	23.7

**Table 3:** The difference in friction and wear response of castor oil with additives compared to the same oil deprived of them.

Ref.	Additive Concentration in *CO*	Lubricating Regime	Test Duration (Minutes)	Friction Response	Wear Response
[13]	8 wt.% of hBN	Mixed-elastohydrodinamic lubrication	10	22% increase	28% decrease
[14]	0.3 v% of TiO_2_	Not specified	60	18% increase	11% increase
[15]	0.7 wt.% of MoS_2_	Not specified	5	12.5% increase	37% decrease
This study	0.001 m of *L*	Boundary lubrication	252	14% increase	15% decrease
This study	0.001 m of *Z*	Boundary lubrication	252	25% increase	8% decrease
This study	0.001 m of *A*	Boundary lubrication	252	22% increase	42% decrease
This study	0.86 v% of ZDDP	Boundary lubrication	252	23% decrease	31% increase

## Data Availability

The data presented in this study are available on request from the corresponding author.
